# Combination Therapy in Fragile X Syndrome; Possibilities and Pitfalls Illustrated by Targeting the mGluR5 and GABA Pathway Simultaneously

**DOI:** 10.3389/fnmol.2017.00368

**Published:** 2017-11-07

**Authors:** Shimriet Zeidler, Helen de Boer, Renate K. Hukema, Rob Willemsen

**Affiliations:** Department of Clinical Genetics, Erasmus University Medical Center, Rotterdam, Netherlands

**Keywords:** Fragile X syndrome, *FMR1*, GABA, bumetanide, mGluR5, automated tube test, autism, *Fmr1* KO mouse

## Abstract

Fragile X syndrome (FXS) is the most common monogenetic cause of intellectual disability and autism. The disorder is characterized by altered synaptic plasticity in the brain. Synaptic plasticity is tightly regulated by a complex balance of different synaptic pathways. In FXS, various synaptic pathways are disrupted, including the excitatory metabotropic glutamate receptor 5 (mGluR5) and the inhibitory γ-aminobutyric acid (GABA) pathways. Targeting each of these pathways individually, has demonstrated beneficial effects in animal models, but not in patients with FXS. This lack of translation might be due to oversimplification of the disease mechanisms when targeting only one affected pathway, in spite of the complexity of the many pathways implicated in FXS. In this report we outline the hypothesis that targeting more than one pathway simultaneously, a combination therapy, might improve treatment effects in FXS. In addition, we present a glance of the first results of chronic combination therapy on social behavior in *Fmr1* KO mice. In contrast to what we expected, targeting both the mGluR5 and the GABAergic pathways simultaneously did not result in a synergistic effect, but in a slight worsening of the social behavior phenotype. This does implicate that both pathways are interconnected and important for social behavior. Our results underline the tremendous fine-tuning that is needed to reach the excitatory-inhibitory balance in the synapse in relation to social behavior. We believe that alternative strategies focused on combination therapy should be further explored, including targeting pathways in different cellular compartments or cell-types.

## Introduction

Fragile X syndrome (FXS) is a common X-linked hereditary cause of intellectual disability and autism spectrum disorders (ASD), with a prevalence of about 1:7000 males and 1:11,000 females (Coffee et al., 2009; Hunter et al., 2014). FXS is mainly characterized by cognitive and behavioral symptoms (Garber et al., 2008; Hersh et al., 2011; Kidd et al., 2014; Lozano et al., 2014). The autistic behavior and social deficits lead to major disabilities and are important features of FXS to evaluate when testing efficacy of potential pre-clinical therapeutic interventions. FXS is currently treated symptomatically, using behavioral, educational and psychopharmaceutical strategies, often with unsatisfying results. A targeted treatment is lacking.

Since the discovery of the *FMR1* gene as the causative gene of the disease, and the generation of the *Fmr1* KO mouse model, research has focused on elucidating the molecular basis of the disorder. The discovery of several pathways involved has revealed possible targets for therapeutic intervention strategies, holding the promise for a disease modifying therapy. Targeting these pathways indeed could correct many FXS-related symptoms in animal models, however, these promising preclinical results could not be confirmed in clinical trials (reviewed in Braat and Kooy, 2014; Ligsay and Hagerman, 2016). Many reasons could explain this lack of translation from mice to human (Zeidler et al., 2015). One striking limitation in drug discovery research so far, is the oversimplification of the underlying molecular mechanisms of the disorder, by targeting only one pathway at a time. The vast amount of molecular targets of the *FMR1* gene product, FMRP, suggests that the use of a combination therapy, targeting multiple involved pathways simultaneously, is a promising new strategy in drug discovery for FXS. In this article we discuss the possible use of combination therapy in FXS. In addition, we present the first *in vivo* data on chronic combination therapy, targeting both the excitatory and inhibitory system in the synapse in *Fmr1* KO mice. Our data illustrate that the two synaptic pathways are interconnected, although tremendous fine-tuning is probably required to restore the synaptic excitatory/inhibitory balance.

## Many Targets, Many Drugs

The symptoms of FXS are caused by lack of FMRP, an RNA-binding protein that plays a critical role in the process which determines neuronal connectivity, called synaptic plasticity (Willemsen et al., 2011). In the *Fmr1* knock-out (*Fmr1* KO) mouse this synaptic plasticity is disrupted, leading to neuronal dysfunction. Several pathways are implicated in aberrant synaptic plasticity in FXS, revealing them as possible targets for therapy. The metabotropic glutamate receptor 5 (mGluR5) pathway and the γ-Aminobutyric acid (GABA) pathway are only two examples (Braat and Kooy, 2014). Many studies have shown that we can indeed target these pathways in the *Fmr1* KO mouse, in some cases leading to improvement of several disease characteristics (reviewed in Braat and Kooy, 2014, 2015; Gross et al., 2015; Scharf et al., 2015). Interestingly, FMRP is not only present in the postsynaptic compartment, but is also expressed in the presynaptic compartment and other cell-types in the brain, although little is known about its function there (Wang et al., 2004; Pacey and Doering, 2007; Christie et al., 2009; Akins et al., 2012, 2017; Giampetruzzi et al., 2013; Higashimori et al., 2013; Gholizadeh et al., 2014). This might implicate more options for targeted therapy.

The mGluR5-pathway was the first proposed and best studied pathway involved in the pathogenesis of FXS, leading in 2004 to the “mGluR5 theory” (Bear et al., 2004). Activation of mGluR5 leads to downstream local protein synthesis in the postsynaptic compartment, which is essential for synaptic plasticity. This local protein synthesis is controlled by FMRP and its absence results in exaggerated mGluR5-dependent protein synthesis and consequently aberrant synaptic plasticity. Several studies have shown that either genetic or pharmacological reduction of mGluR5 restores FXS related phenotypes in *Fmr1* KO mice, including molecular, anatomical, electrophysiological and behavioral characteristics (Dölen et al., 2007; de Vrij et al., 2008; Osterweil et al., 2010; Thomas et al., 2011, 2012; Michalon et al., 2012; Gantois et al., 2013; Pop et al., 2014; Scharf et al., 2015; de Esch et al., 2015). Another important pathway implicated in FXS, is the GABAergic pathway, the major inhibitory pathway in the adult brain (D’Hulst et al., 2006, 2009; Gantois et al., 2006; Curia et al., 2009; Pacey et al., 2009; Adusei et al., 2010; Olmos-Serrano et al., 2010; Sabanov et al., 2017; Zhang et al., 2017). Drugs targeting the GABA_a_ or GABA_b_ receptor, have shown improvements of FXS features in *Fmr1* KO mice. The function of the ionotropic GABA_a_ receptor, a synaptic and perisynaptic chloride channel, can also be indirectly influenced with the Na^+^-K^+^-2Cl^−^-co-transporter 1 (NKCC1) blocker bumetanide (Tyzio et al., 2014). While the GABA_a_ receptor inhibits depolarization in adult neurons, its function in immature neurons during early development is excitatory, switching to inhibitory while the neurons mature. This important neurodevelopmental switch depends on the intracellular chloride levels, regulated by the chloride importer NKCC1 (Ben-Ari et al., 2012; Ben-Ari, 2015). It has been shown to be delayed or absent in *Fmr1* KO mice (He et al., 2014; Tyzio et al., 2014) and FXS derived human embryonic stem cells (Telias et al., 2016). Also in other disorders, a delayed GABAergic switch has been implicated, including autism (Ben-Ari, 2015), epilepsy (Holmes et al., 2015), Parkinson’s disease (Damier et al., 2016) and schizophrenia (Lemonnier et al., 2016). Reduction of chloride levels with bumetanide, forces the neuron to switch from immature to mature chloride concentrations and consequently also to mature GABA_a_ergic function. This has been demonstrated by bumetanide treatment of pregnant mice, which restored electrophysiological and behavioral phenotypes in their *Fmr1* KO offspring (Tyzio et al., 2014). Several clinical trials in patients with autism, have demonstrated improvement after bumetanide treatment (Lemonnier and Ben-Ari, 2010; Lemonnier et al., 2012, 2017; Hadjikhani et al., 2015), rendering it a promising drug in FXS as well.

### Translational Challenges

The promising preclinical results have motivated researchers to initiate clinical trials in FXS patients. Some randomized, placebo controlled clinical trials with the mGluR5 antagonists mavoglurant/AFQ056, fenobam (Berry-Kravis et al., 2009, 2016; Jacquemont et al., 2011) and basimglurant (Youssef et al., 2017) have been performed. However, despite the evidence for effectiveness of mGluR5 antagonists from animal model studies, these clinical trials did not result in improvement of symptoms in FXS patients. Also the larger clinical trials with the GABA_b_ agonist Arbaclofen, were terminated prematurely due to lack of efficacy (Berry-Kravis et al., 2017). In fact, none of the larger clinical trials have resulted in an effective treatment for FXS. This raises the question whether these observed preclinical treatment effects reflect a relevant and versatile treatment strategy. Major limitations that could account for this lack of translation include the lack of reliable and robust outcome measures, aspects of study design and the validity of animal models in drug screening (Berry-Kravis et al., 2013; Zeidler et al., 2015). However, one important aspect is being consistently neglected: considering the vast amounts of targets of FMRP, probably multiple pathways will need to be targeted simultaneously in order to ameliorate the disease, a combination therapy. Current studies in mice as well as in humans have been consistently limited to targeting only one pathway at a time.

## New Strategies in FXS Drug Discovery: Combination Therapy

Compelling evidence has demonstrated that aberrant synaptic plasticity in FXS is (partly) caused by an excitatory-inhibitory imbalance, due to malfunctioning of these pathways (reviewed in Braat and Kooy, 2014; Ligsay and Hagerman, 2016). Thus, we hypothesized that targeting both excitatory and inhibitory pathways simultaneously as combination therapy, might be more beneficial in treating FXS than targeting a single pathway.

Only two previous publications have reported data on combination therapy in *Fmr1* KO mice. Lim et al. (2014) observed a synergistic beneficial effect on synaptic plasticity and behavior when targeting serotonin and dopamine-pathways in *Fmr1* KO mice simultaneously. Pacey et al. (2011b) showed an additional synergistic effect of acute targeting of mGluR5 (MPEP) and GABA_b_ (R-baclofen) in *Fmr1* KO mice on seizures, while for both a lower dose was needed than when administered separately. However, these studies used acute treatment and did not address social behavior deficits. Especially when initiated later in life, treatment of FXS would probably require a life-long treatment. To our knowledge we are the first to investigate the effect of chronic combination therapy in *Fmr1* KO mice, and using social behavior as an outcome measure.

**Figure 1:**
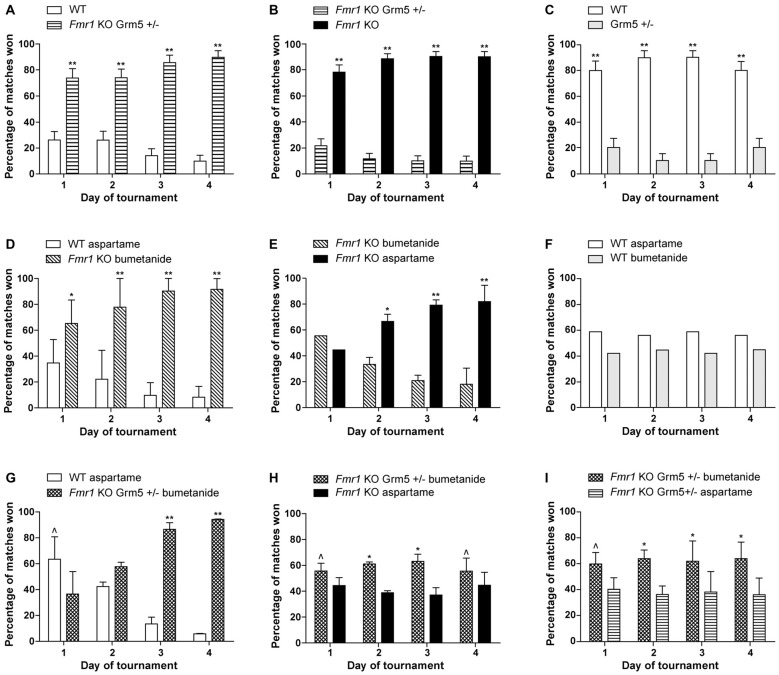
Reduction of metabotropic glutamate receptor 5 (mGluR5) or enhancing γ-aminobutyric acid (GABA) separately, partially improves the Fragile X syndrome (FXS) phenotype, while a combination therapy slightly worsens this effect. Results are indicated as percentage of matches won by *Fmr1* KO mice and wild-type (WT) littermates. To explain what is meant by a partial correction in the tube test: a full correction would implicate a 50%–50% result of the matches between WT mice and treated *Fmr1* KO mice. In that case, both groups show a similar social behavior phenotype. When a partial correction is observed, there is a clear dominant phenotype of untreated *Fmr1* KO mice compared to treated *Fmr1* KO mice, while treated *Fmr1* KO mice do not show a correction in matches against WT mice. **(A–C)** Previously published results from de Esch et al. (2015) presenting that genetic reduction of mGluR5 partially corrects the automated tube test (ATT) phenotype in *Fmr1* KO mice. **(A)** *Fmr1* KO mice who are *Grm5*+/− continue to show a strong phenotype compared to their WT littermates (*p* < 0.001, *n* = 12 per group). **(B)** Strong reduction of ATT phenotype with mGluR5 reduction: *Fmr1* KO win most matches against *Fmr1* KO mice who are *Grm5*+/− (*p* < 0.001, *n* = 10 per group). **(C)** Genetic reduction of mGluR5 induces an inverse phenotype in the WT animals in the ATT (*p* < 0.001, *n* = 6 per group). **(D–F)** Chronic treatment with bumetanide partially correct the ATT phenotype in *Fmr1* KO mice. **(D)** *Fmr1* KO mice treated with bumetanide continue to show a strong phenotype compared to their WT littermates receiving aspartame drinking water (*p* < 0.01 on day 1 and *p* < 0.001 on day 2–4, *n* = 12 per group). **(E)** Strong reduction of ATT phenotype after bumetanide treatment comparing treated and untreated *Fmr1* KO mice: *Fmr1* KO receiving aspartame water win most matches against *Fmr1* KO treated with bumetanide after day 1 (*p* < 0.01 on day 2, *p* < 0.001 on day 3 and 4, *n* = 12 per group). **(F)** WT mice receiving bumetanide in their drinking water and WT mice receiving aspartame drinking water win equal amounts of matches (*p* > 0.1 for all days, *n* = 6 per group). **(G–I)** Combination of genetic mGluR5 reduction and bumetanide treatment results in a slight worsening of the ATT phenotype compared to mGluR5 reduction or bumetanide treatment alone. **(G)** *Fmr1* KO who are *Grm5*+/− and treated with bumetanide lose most matches against WT receiving aspartame drinking water on day 1 (*p* = 0.02) but win most matches on day 3 and 4 (*p* < 0.001, *n* = 10 mice per group). **(H)** *Fmr1* KO mice who are *Grm5*+/− and treated with bumetanide win slightly more matches than* Fmr1* KO receiving aspartame drinking water (*p* = 0.04 on day 1 and 4 and *p* < 0.01 on day 2 and 3, *n* = 18 mice per group). **(I)** *Fmr1* KO who are *Grm5*+/− and treated with bumetanide win slightly more matches than *Fmr1* KO *Grm5*+/− receiving aspartame drinking water (*p* = 0.01 to *p* = 0.002, *n* = 17 mice per group). Data shown as mean percentage ± SEM. *P*-values were calculated using a binomial distribution test was: in an experiment, both groups are similar if approximately 50% of matches are won per group, **<0.001, *<0.01, ^<0.05.

The results of our combination therapy experiments are depicted in Figure 1. We targeted the mGluR5 pathway by genetically reducing mGluR5 expression, and the GABAergic pathway using the commercially available diuretic bumetanide. These pathways were first targeted separately (Figures 1A–F) and then simultaneously (Figures 1G–I). We used *Fmr1* knock-out mice (Mientjes et al., 2006), their wild-type (WT) littermates and for the double transgenics, we crossed these with mice who were heterozygous for an mGluR5 deletion (*Grm5*+/−; Lu et al., 1997). We measured the effect of the therapeutic interventions using a social behavior paradigm, the automated tube test (ATT). The protocols are extensively described in de Esch et al. (2015) and van den Berg et al. (2015). Mice received bumetanide (Centrapharm) dissolved in drinking water in a concentration of 0.01 mg/ml, based on Tyzio et al. (2014), and kept in light-tight bottles. Aspartame was added to reduce the bitter drug taste. Control mice of the experiments with bumetanide, received aspartame drinking water. Control drinking water containing aspartame has been shown to have no effect on the *Fmr1* KO phenotype in the ATT (data not shown). Mice were chronically treated from weaning at postnatal week 4 until the end of the experiment, postnatal week 13–16. This study was carried out in accordance with the recommendations of Directive 2010/63/EU, European Commission. The protocol was approved by the Dutch Animal Ethical Committee (DEC).

Previously, we have published that *Fmr1* KO mice display a robust dominant ATT phenotype compared to WT littermates, resulting in significantly increased percentage of matches won by *Fmr1* KO mice (de Esch et al., 2015). Figures 1A–C display the results of previously published experiments, showing that genetic reduction of mGluR5 results in a partial correction of social behavior of *Fmr1* KO mice in the ATT (de Esch et al., 2015). A complete correction would lead to a 50%–50% distribution of the matches between WT and *Fmr1* KO mice. The correction is partial, since no change in the phenotype is observed in those matches, after genetic reduction of mGluR5 in the *Fmr1* KO animals (Figure 1A). However, compared to “untreated” *Fmr1* KO mice, they do lose their phenotype (Figure 1B), illustrating the treated mice do no longer behave as *Fmr*1 *KO* mice. If there would have been no effect of treatment, a 50–50 distribution of wins over the two groups was expected. This partial correction indicates that targeting the mGluR5 pathway does significantly influence the social behavior phenotype, but is not sufficient to fully restore deficits in this type of social behavior. A quite similar effect was observed when targeting the GABAergic pathway, using chronic bumetanide treatment. Figures 1D–F depict the results of chronic bumetanide treatment, leading again to a partial correction of the FXS ATT phenotype. These results indicate that treatment with bumetanide by itself is insufficient as well. However, these results do underline that bumetanide might have a beneficial effect on social behavior in FXS patients. Since we administered bumetanide after the GABAergic developmental switch has occurred (He et al., 2014), the improvement we measure is encouraging in terms of treatment initiation later in life, although the exact underlying neurochemistry changes remain to be elucidated.

After the partial correction observed for both “treatment interventions” separately, we combined those. However, combination therapy leads to an opposite effect than expected. First, the *Fmr1* KO mice with combination therapy remain dominant in matches against WT animals (Figure 1G). Moreover, *Fmr1* KO mice with combination therapy show a mild but significant dominant phenotype against “untreated” *Fmr1* KO mice (Figure 1H), implicating worsening of the phenotype. Improvement of the phenotype would lead to dominant behavior of untreated mice, which is opposite to what we observed. To evaluate whether a subtle synergistic effect occurs with two treatments compared to one treatment alone, we performed the test comparing *Fmr1* KO mice with either mGluR5 reduction alone to *Fmr1* KO mice with a combination of mGluR5 reduction and bumetanide. A synergistic effect would have led to dominant behavior of *Fmr1* KO mice with one intervention, compared to those with a combination therapy. However, we did not observe a synergistic effect, but instead we observed a slight worsening of the ATT phenotype in *Fmr1* KO mice with a combination therapy, compared to one intervention alone (Figure 1I). This might be explained as an antagonistic effect. Although no synergy was observed, clearly targeting the two pathways simultaneously, does create a combined effect, attenuating their therapeutic efficacy on FXS social behavior deficits, and confirming the pathways are interconnected.

## Optimal Window in Combination Therapy

**Figure 2:**
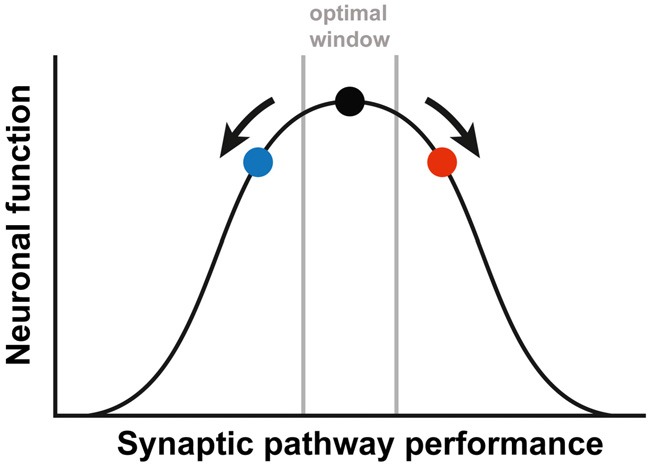
A simplified depiction of the relationship between the synaptic pathway performance and the neuronal function. The black dot represents the optimal function, as is the case in WT animals. Either increased (red dot) or decreased (blue dot) performance leads to a suboptimal function of the neuronal synapses. In order to correct FXS, therapy needs to be fine-tuned, to prevent an overshoot (going from the red to the blue dot) or a worsening of the synaptic pathway performance (going from the red dot further to the right). The figure is based on the article by Auerbach et al. (2011).

Interestingly, the partial rescue that we observed for both treatments separately, is reduced when they are combined, even leading to a slight worsening of the phenotype. These results implicate that the treatment effect might be managed by adding different interventions and titrating those to reach an optimal effect. It has been previously suggested by Auerbach et al. (2011) that synaptic plasticity is a tightly regulated process. The authors demonstrated an optimal window for protein synthesis levels. Deviations to either side of this optimum, resulted in decreased functioning of the synapse and aberrant synaptic plasticity. This idea of an optimal synaptic function due to a balanced interconnection of involved pathways, could be generalized to the excitatory/inhibitory balance of the synapse or to synaptic performance in general. Considering this optimum, our results might be explained by either an opposing effect of both treatments, or by an overshoot effect of both treatments when combined (Figure 2). In both cases, this means that restoring this tightly regulated balance will need tremendous fine-tuning. Unfortunately, it is poorly understood how the mGluR5 and GABAergic pathways are interconnected at the synapse and no biochemical read-out is available to test whether the right balance has been reached (Martin and Huntsman, 2012; Fatemi and Folsom, 2015). To complicate matters, the required balance might be significantly different in different brain regions or even differ at the synaptic level within one neuron, since FMRP is not localized in every spine (Feng et al., 1997; Antar et al., 2004).

Obviously, the results presented in Figure 1 regard only one specific social behavior paradigm, which does not inform us on the effect on other FXS behavioral and cognitive phenotypes. In addition, only two pathways were targeted. Possibly, other pathways are more important for social behavior and targeting those might result in a beneficial effect. Even though we currently cannot demonstrate a synergistic effect, it seems plausible that targeting only one pathway is not sufficient to ameliorate FXS completely. While considering the lack of translation from mice to human, combination therapy has received little attention, even though we are only starting to grasp the complex role of FMRP in synaptic plasticity. FMRP binds many post-synaptic mRNAs, that are involved in important neuronal synaptic pathways. Moreover, FMRP does not only have a postsynaptic function, but is also present in the presynaptic compartment and other cell types, including glia cells (Wang et al., 2004; Pacey and Doering, 2007; Pacey et al., 2011a; Giampetruzzi et al., 2013; Higashimori et al., 2013; Myrick et al., 2015). Thus, absence of FMRP potentially disrupts many cellular pathways, each with its own function. Recently, a missense mutation in *FMR1* has been identified in a patient, demonstrating a specific function of FMRP in the presynaptic compartment (Myrick et al., 2015). The patient only displayed ID and seizures, but did not display the behavioral problems associated with FXS, suggesting different pathways in different cellular compartments might be associated with specific FXS symptoms and phenotypes. Additionally, FMRP is present in other non-neuronal cell-types, where its function is even less understood (Wang et al., 2004; Pacey and Doering, 2007; Higashimori et al., 2013). For example, compelling evidence demonstrates the role of astrocytes, in neuronal maintenance, but also in active control of synaptic function, leading to the new concept of the tripartite synapse (Cheng et al., 2012). FMRP is present in the astrocytes, and its absence has been demonstrated to hamper normal astrocyte function, opening a new field of possible therapeutic strategies. An additional reason that advocates combination therapy, is the presence of compensational mechanisms that add to the individual differences. Targeting more than one unit of a pathway could be more effective and specific, with a lower dose needed, reducing the chance for side effects.

Other research fields have a longer history of combining targeted treatments to improve therapy. For example, studying the complex genetics of cancer has led to the identification of key-oncogenic cellular pathways, enabling the use of a combination of targeted pharmacological treatments to selectively block and kill tumor cells (Yap et al., 2013). However, these settings often have access to high throughput study models in cell culture and well-defined outcome measures, which are lacking in neurodevelopmental research. In recent years, combination therapy in neurodevelopmental syndromes have been proposed, for example in Rett syndrome (Sahin and Sur, 2015) and tuberous sclerosis complex (Lee et al., 2006). In FXS patients, one case report mentioned combination therapy with two drugs in combination with intensive educational treatment in two children, resulting in improvement of cognition and behavior (Winarni et al., 2012). In the near future, a clinical trial treating FXS patients with a combination of lovastatin and minocycline, will start (NCT02680379). New pre-clinical studies are needed to further evaluate the role of FMRP in other cell-types and to reveal new targets for therapy. Those targets should be used to investigate whether combination therapy is the key solution for FXS treatment, by targeting multiple pathways in different cellular compartments or cell-types. Probably, all those interventions must be applied in combination with stimulating behavioral and cognitive therapy, to maximize therapeutic effects.

## Conclusion

In conclusion, the complexity of the pathophysiology of FXS and the lack of translation from mouse to human, indicates that combination therapy is essential in the development of a targeted therapy for FXS syndrome. This approach needs to be further explored and might become successful, using other drugs, or targeting pathways in different cellular compartments, for example pre- and postsynaptic, or even other cell-types. However, combination therapy will need to be fine-tuned, in order to restore the tightly regulated synaptic pathway balance.

## Author Contributions

RW has been involved in design of experiments, supervising PhDs, correcting draft manuscript. SZ has been involved in performing experiments, design of experiments, interpretation of results, statistics, writing draft manuscript. HB has been involved in performing experiments and interpretation of results, statistics, correcting draft manuscript. RKH has been involved in supervising PhDs, design of experiments, correcting draft manuscript.

## Conflict of Interest Statement

The authors declare that the research was conducted in the absence of any commercial or financial relationships that could be construed as a potential conflict of interest.
